# Monthly Summary

**Published:** 1889-04

**Authors:** 


### Monthly Summary.

What Shall We Eat?—When I examine the teeth of those who live in the cold regions, as the Esquimaux, who live entirely on a fatty diet, I find them of good character and structure; and as I pass to other regions, where the people live entirely on a vegetable and a fruit diet, I find their teeth of the same general character as those of the meat and fat eaters. Also, when we examine the teeth of the early races of this continent, or in any quarter of the globe, we find the same character of teeth associated with the greatest variety of food habit. The question arises whether a meat diet or a vegetable diet is best for the human race. I hold that we may eat almost any form of food, but that we are not built for a meat diet. This is my positive conviction. I know it is contrary to general opinion, and the shape of the teeth, especially of the canines, is cited in opposition to it. It is a fact that the human animal has comparatively smaller teeth than many of the herbiverous animals; that does not indicate a meat diet. We now live on a mixt diet, but I think the time will come when the human race will refuse to eat anything that "has just died," as Dr. Atkinson puts it. To my mind the whole human race is gradually advancing from a coarse stage of existence, from a time when the flora and fauna on the earth's surface were rude and coarse. Yet that is an expression which we cannot properly use in this connection, because there is nothing coarse or fine in nature, but still there is a difference in the grain, not only in organized beings, but in vegetables that then existed; they were coarser in grain and coarser of organization than those of the present, and probably those of the future; and the reason, I think, lies in the fact that the food of those animals and vegetables was in a comparatively coarse and crude condition. The earth has been going through a refining process, as it were, and we find a different race of animals and a different race of men; and this change will go on till that condition comes when the earth will be peopled by a race of men and women who will not only not eat meat, but will probably be so spiritually improved by the better food taken into their systems that they will be an entirely different race from that which now exists. I believe that all nature points to that result. I believe that not only this earth, but the great series of worlds, every one of which is, or will be, peopled similarly to ours, must eternally advance toward the good in the refinement and elevation of the races which exist on them. *—Dr. James Truman, Philadelphia.*

